# Notes and News

**Published:** 1900-08-18

**Authors:** 


### Notes and News.

A sanatorium for tuberculosis is to be opened on August 23rd at Hauteville-en-Bugey, France, by the Lyons Association for the Tuberculous Poor.

Cholera continues to spread in India, and it is unfortunately largely affecting British and native troops, and continues to interfere with their embarkation for China.

At the International Congress of Medicine in Paris still another cure for tuberculosis has been put forward, founded on the direct transmission of antiseptics by means of static electricity.

August 24th being the celebration of Professor Virchow's golden wedding, his scientific friends are taking the opportunity to express their admiration by means of suitable congratulations. All the German scientific societies are sending their congratulations.

The expeditions which the Liverpool School of Tropical Medicine has sent out to Africa and America have sent back an account of their doings. The American expedition seems to have been most hospitably received. At the request of Dr. Sternberg, the members have gone to Cuba with the expedition of the American Government to study yellow fever in Havana.

As a result of a visit of the Commissioners to the Harrisburg Insane Asylum, U.S.A., it has been recommended that many patients sent there might well be retained at home, as not violent, and capable of being cared for by their friends. It is quite natural that those responsible for the expenditure of public moneys on the insane should carefully check abuse, but it is a retrograde step to advise the retention of the weak-minded and irresponsible amongst that class of the community where they can do most harm and be most neglected. In England the care of the feeble-minded is being recognised not only as a charity, but as an important economic step towards social progress. It is to be hoped that if stricter lines are drawn for the reception of patients at Harrisburg that some other provision will be made for the protection of the weak-minded who cannot find a shelter in the asylum.

We learn that a church in Ipswich is the first to lead the way in England towards banishing the objectionable system of a common cup for the congregation when celebrating Holy Communion. The adoption of individual cups is certainly a more seemly as well as hygienic plan.

The Ladies' Committee of the Hospital Ship "Maine," which is on its way to China, are appealing again to American women especially to support it, but all contributions will be welcomed, as the ship is a Red Cross vessel, destined for the use of the wounded of all nationalities. Some American ladies have already contributed large sums.

Extraordinary instances of poisoning by post are coming from Paris. It appears that a Polish lady has been sending envelopes, apparently without contents, or with some small object inside, addressed to members of various foreign legations principally. The recipients have been seized with sudden illness, and the lady has been arrested.

The heat is again claiming its victims after the spell of cold weather which has intervened since the last heat wave. Several men have already fallen victims to the great heat of the sun, and horses are suffering very much. The straw hats, which are so largely adopted by London owners for their horses, have proved very successful in mitigating the effect of the fierce sun. There is a large amount of unripe fruit in the London markets at the present time, and it is not unlikely that a good deal of sickness will result.

The cause of the disabled soldier at home and abroad keeps controversialists very busy. Letters continue to flood the newspapers in respect to the methods of the Army Medical Service, the conditions of the hospitals in South Africa, and the failings of the Commission of Inquiry. Letter-writers are equally busy discussing the provision for the disabled soldier at home on his return from the war. Unfortunately the origin of the scheme for the home at Bisley is not clear, nor the authority under which it works, consequently columns of type are now devoted to discussing the question.

The Royal Institute of Public Health has held its congress in Aberdeen. Between eight and nine hundred delegates attended. The inaugural address was delivered by the Earl of Aberdeen. The Presidents of the various sections afterwards delivered their addresses. Professor W. Simpson, of King's College, spoke on Administration and Research in Preventive Medicine. He especially called for an Imperial and Colonial Sanitary Service. Professor D. Hamilton, of Aberdeen, discussed recent developments in bacteriology; Professor Hunter Stewart, of Edinburgh, delivered his address upon Meteorological Conditions and Periodical Epidemic Diseases; Mr. John Honeyman, of Glasgow, dwelt on the structure of dwelling-houses, and the importance of rendering the actual site of such sanitary before building. Dr. R. Farquharson, M.P., devoted his remarks to the subject of Municipal and Parliamentary Hygiene. The addresses were followed by discussions, demonstrations, and social functions, and terminated on August 7th.

